# Does AI-assisted labor change how earnings are spent?

**DOI:** 10.3389/fpsyg.2026.1877013

**Published:** 2026-09-03

**Authors:** Jinru Zong, Zhuo Lyu

**Affiliations:** 1 International Cultural and Educational College, Northeast Agricultural University, Harbin, China; 2 School of Economics and Management, Northeast Agricultural University, Harbin, China

**Keywords:** artificial intelligence, effort heuristic, mental accounting, perception–behavior gap, psychological ownership

## Abstract

Generative AI is rapidly substituting for human cognitive effort in everyday labor. This substitution attenuates the cues that make income feel earned: subjective effort and felt ownership of the work product. Mental-accounting theory suggests that earnings stripped of these cues should be treated more like windfalls than like earned income. In a preregistered between-subjects experiment, participants completed a real-effort copywriting task under *Manual, Augmented*, or *Substituted* AI assistance and then made an incentive-compatible investment decision over their earnings. The manipulation produced large monotone shifts in every perceptual measure (subjective effort, psychological ownership, perceived self-contribution, and AI attribution), including the feeling that the reward was earned. None of the preregistered hypotheses was supported: investment allocation and hedonic–utilitarian consumption choice showed no significant condition differences (nor did self-rated risk preference, a secondary outcome), and the preregistered bootstrap indirect effects through effort and ownership all included zero. The behavioral point estimates were directionally consistent with the predictions but far too imprecise to adjudicate them, falling well below the study's minimum detectable effect, so the data bound medium-to-large effects rather than establish absence. A categorical mental-accounting classification of the earnings did shift toward “windfall,” but its anchors partly restate the effort manipulation, so this shift should not be read as an independent behavioral finding. We interpret the results as strong evidence that AI assistance changes the felt effort, authorship, and earnedness of income, and as quantitative bounds on, rather than proof against, the downstream spending consequences that mental-accounting theory predicts.

## 1 Introduction

Generative artificial intelligence (GenAI) is rapidly reshaping what counts as cognitive work. Field and experimental studies report substantial productivity gains across a range of cognitive work, including customer-service operations (Brynjolfsson et al., 2025), consulting and knowledge work (Dell'Acqua et al., 2023), and mid-level professional writing (Noy and Zhang, 2023). Because these productivity gains come from substituting *human cognitive effort* rather than physical capital or time, they raise a question that matters well beyond the laboratory: once the cognitive effort has been outsourced, is the resulting income still treated as “earned”?

This question matters for at least two reasons. First, classical mental-accounting theory predicts that income represented as more windfall-like and less earned should be treated differently in risk taking, savings, and hedonic consumption (Thaler, 1985, 1999). Second, a rapidly growing literature on AI at work infers behavioral consequences from *perceived* changes in meaningfulness, effort, or ownership (Jussupow et al., 2020; Tang et al., 2022). Whether those self-reported perceptions translate into downstream economic decisions is an open question that can only be settled by experiments pairing perceptual measures with real-stakes behavior. We stress at the outset the scale at which the present experiment can speak to these questions: it tests the perception-to-behavior link over a small, one-shot laboratory task, and none of its preregistered behavioral hypotheses were supported. Any extrapolation to labor-market or policy-level consequences would require field variation in earnings of consequential size and is beyond what this design can license.

Existing work on AI in the workplace has largely split between two frames: the labor-market consequences of AI adoption, and attitudes toward AI, including algorithmic aversion and appreciation (Dietvorst et al., 2015; Logg et al., 2019). The question we take up here falls between them. Holding the monetary outcome fixed, does the intensity of AI assistance during the work change how the resulting income is classified and spent?

Mental-accounting theory (Thaler, 1985, 1999) holds that people do not treat money as fungible but rather partition it into “accounts” defined in part by the income's psychological origin. Windfall income, defined as money perceived as unearned, unexpected, or otherwise external to the self, is more readily spent on hedonic goods (Arkes et al., 1994; Epley and Gneezy, 2007; O'Curry, 1999), allocated toward risky investments (Thaler and Johnson, 1990), and disposed of less cautiously than income classified as earned (Henderson and Peterson, 1992; Lea and Webley, 2006). Earned income, by contrast, is money tied to one's own effort, competence, or identity, and tends to be handled with greater self-regulation, caution, and goal-consistency. A parallel experimental-economics literature manipulates the earned–windfall status of endowments directly and finds that earned money is treated differently, most reliably in allocations to others (Cherry et al., 2002; Clark, 2002; Jakiela, 2015); that literature informs both the payment structure that a strong test requires, and the outcome domains in which earnedness effects are best documented.

Where does AI-assisted earning fall on this earned–windfall continuum? Windfall status has several distinguishable cues: unexpectedness, external origin, and low own effort (Arkes et al., 1994; Thaler, 1999). AI assistance leaves the first two untouched, since in every condition of our design, participants know they will be paid and the money arrives for their task, and operates specifically on the third: it dilutes the subjective effort invested in producing the output and the sense that the output is self-authored. When AI generates or heavily prestructures the product of one's labor, the experiential correlates of earning it (cognitive strain, authorship, creative investment) are attenuated, even when the monetary payoff and the nominal work role are held constant. Work on the value-conferring role of labor makes the same point from the production side: self-made products are valued more (Norton et al., 2012), and visible effort raises perceived value even when output is held constant (Buell and Norton, 2011; Kruger et al., 2004). Our manipulation is therefore best understood as targeting the *earnedness* cue of windfall status, not its unexpectedness; whether moving earnedness alone suffices to shift spending behavior is precisely what the experiment tests.

This predicted shift implies two behavioral outcomes. If AI-assisted earnings are mentally accounted closer to a windfall, participants should (a) be more willing to place that money at risk, a direct extension of the house-money effect (Arkes et al., 1994; Thaler and Johnson, 1990), and (b) allocate more of it toward hedonic rather than utilitarian consumption (Levav and McGraw, 2009; O'Curry, 1999). Additionally, the categorical classification of the income on an earned–windfall scale should itself shift. We refer to this integrated prediction as the *AI-windfall hypothesis*. We note, however, that the directional predictions are not theoretically unambiguous. If AI-assisted earnings feel tainted or illegitimate rather than merely unearned, emotional-accounting work implies the opposite consumption prediction: negatively valenced money is preferentially “laundered” through utilitarian rather than hedonic spending (Levav and McGraw, 2009). On risk, windfall accounting predicts more risk-taking, but reduced felt competence over the AI-produced output could plausibly predict less. A null result can therefore arise either from the absence of any effect or from cancellation between opposing channels; we return to this in the Discussion.

**Figure 1:**
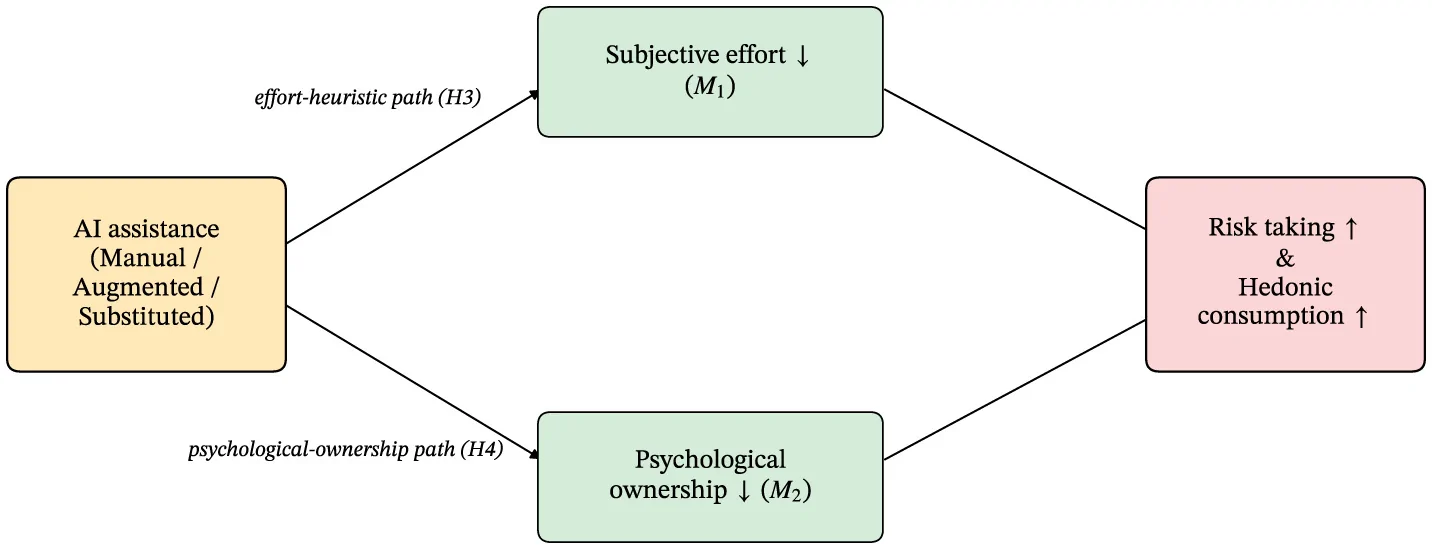
Dual-pathway mediational model as tested. AI assistance is hypothesized to affect downstream risk and consumption behavior through two parallel mediators: subjective effort (*M*_1_; the effort-heuristic path, H3) and psychological ownership (*M*_2_; the psychological-ownership path, H4). The theorized links behind each path (reduced effort lowering the felt earned quality of the money; reduced ownership weakening identity-consistent guarding of the earnings) are developed in the text and are not separately measured constructs in the tested model.

Through what mechanism would AI assistance carry this predicted effect from manipulation to behavior? We test two complementary mediational pathways grounded in classical social-cognitive research, summarized in Figure 1.

The first pathway follows the *effort heuristic* (Kruger et al., 2004): perceived effort serves as a heuristic cue for the value and earned quality of an outcome, a link replicated across domains from artistic appraisal to gift valuation (Aronson and Mills, 1959; Morewedge et al., 2009). Applied here, AI assistance reduces the subjective effort participants feel they invested, which in turn reduces the perceived quality of the money and frees it to be treated more loosely (hypothesis H3). The second pathway follows *psychological ownership* (Pierce et al., 2003), a state produced by controlling the target, investing the self in it, and coming to know it intimately. AI assistance partially interrupts each of these routes (control is shared with the AI, self-investment is diluted by AI pre-structuring, intimate familiarity is replaced by surface interaction with AI-generated options), and lower ownership has been linked downstream to reduced identity-consistent guarding of the owned object (Avey et al., 2009) (hypothesis H4).

The two pathways share an antecedent (AI assistance) and a consequent (changed risk or consumption behavior) but differ in psychological content: effort is a process-level attribution (how hard did I work?), ownership is a product-level attribution (is this mine?). We therefore model them as parallel mediators, allowing each to carry an independent share of the total effect and to be tested against the other.

We report a between-subjects experiment (main confirmatory sample *n* = 179; *N* = 202 with a pre-preregistration pilot batch pooled as robustness) in which participants completed three rounds of a real-effort copywriting task under one of three AI-assistance conditions: *Manual* (unassisted), *Augmented* (AI generates three candidate headlines per product; participant selects and freely edits), or *Substituted* (AI generates and automatically selects). All three conditions yielded identical task earnings of 10 RMB, every participant being subsequently paid the actual outcome of an incentive-compatible investment gamble (range 0–25 RMB) over those earnings. After the copywriting task, participants completed a one-shot investment gamble modeled on (Gneezy and Potters 1997), a binary hedonic–utilitarian consumption choice, a self-rated risk-preference item, subjective-effort and psychological-ownership scales, contribution-attribution items, mental-accounting categorization items, an independent staircase-based risk-preference task, and standard demographic items. Condition assignment was randomized at the individual level within each session.

Using Helmert contrasts (*D*_1_ capturing the *Manual* vs. AI-pooled contrast and *D*_2_ capturing the *Augmented* vs. *Substituted* contrast), we stated the following primary hypotheses *a priori*:

- **H1a**: Investment allocation increases from *Manual* to AI-assisted conditions (*D*_1_>0).
- **H1b**: Investment allocation increases further from *Augmented* to *Substituted* (*D*_2_>0).
- **H2a**: Hedonic consumption is more likely in AI-assisted than in *Manual* conditions (*D*_1_>0).
- **H2b**: Hedonic consumption is more likely under *Substituted* than under *Augmented* (*D*_2_>0).
- **H3**: The effect of AI assistance on investment is mediated by subjective effort.
- **H4**: The effect of AI assistance on investment is mediated by psychological ownership.

A parallel descriptive prediction was also formulated for the categorical mental-accounting classification of the earnings: AI-assisted earnings should be classified closer to the windfall pole than *Manual* earnings. All primary hypotheses, exclusion rules, analytic models, and bootstrap specifications were fixed in a data-processing plan before full-sample analysis. To anticipate the results: none of the six preregistered hypotheses (H1a–H4; four behavioral, two mediational) was supported, while the manipulation produced large and monotone effects on every perceptual measure. The contribution of the article is accordingly the demonstration that AI assistance reliably changes the felt effort, ownership, and earnedness of income, together with quantitative bounds on how large any downstream behavioral consequence can be at this stake size.

## 2 Materials and methods

### 2.1 Experimental design and procedure

The experiment was programmed in oTree (Chen et al., 2016) and conducted online in scheduled group sessions: participants joined a Tencent Meeting video room at the appointed time, opened the experiment link in their browser, and completed the procedure before leaving the room. The study used a one-way between-subjects design with three levels of AI assistance: *Manual, Augmented*, and *Substituted*. The manipulation was administered through three rounds of a real-effort copywriting task, each round asking participants to produce a one-line promotional headline for a different consumer product (Bluetooth earphones, a vacuum flask, and a portable charger; product brand, descriptive specifications, and retail price were displayed on screen). The task instruction was identical across conditions: “Your task is to produce a one-line promotional headline for this product.” The three conditions graded the depth of human contribution to the output:

- *Manual*: participants typed the headline themselves into a text box, without any AI suggestions.
- *Augmented*: participants entered three product-relevant keywords; an LLM-based system^1^ returned three candidate headlines conditioned on those keywords; participants selected one candidate and could freely edit the selection before submission.
- *Substituted*: participants clicked an “auto-generate” button; the LLM-based system returned a single candidate headline for the product, which became the submitted output upon confirmation. Participants did not select among options or edit the text.

All three conditions yielded the same fixed task earnings of 10 RMB upon completion, holding the realized monetary outcome constant so that any downstream behavioral difference cannot be attributed to wealth or scale effects. Two qualifications apply. First, a standing notice (constant across conditions) announced quality bonuses of up to 50 RMB for the top-rated headlines, so *expected* total earnings, and their subjective variance, were not strictly identical in participants' minds; in particular, *Substituted* participants had less basis for appraising the output on which the bonus depended. Second, the consent page disclosed the 10 RMB completion fee before the manipulation, and payment was not contingent on output quality, which works against an “earned-through-effort” construal even in the *Manual* condition; we return to both points in the Discussion. After each round, a brief feedback screen reinforced the condition-specific authorship attribution by displaying a condition-specific badge above the participant's submitted headline (full wording in Appendix 1). After the third round, a reward-notification screen confirmed the 10 RMB earnings with condition-specific framing.

**Figure 2:**
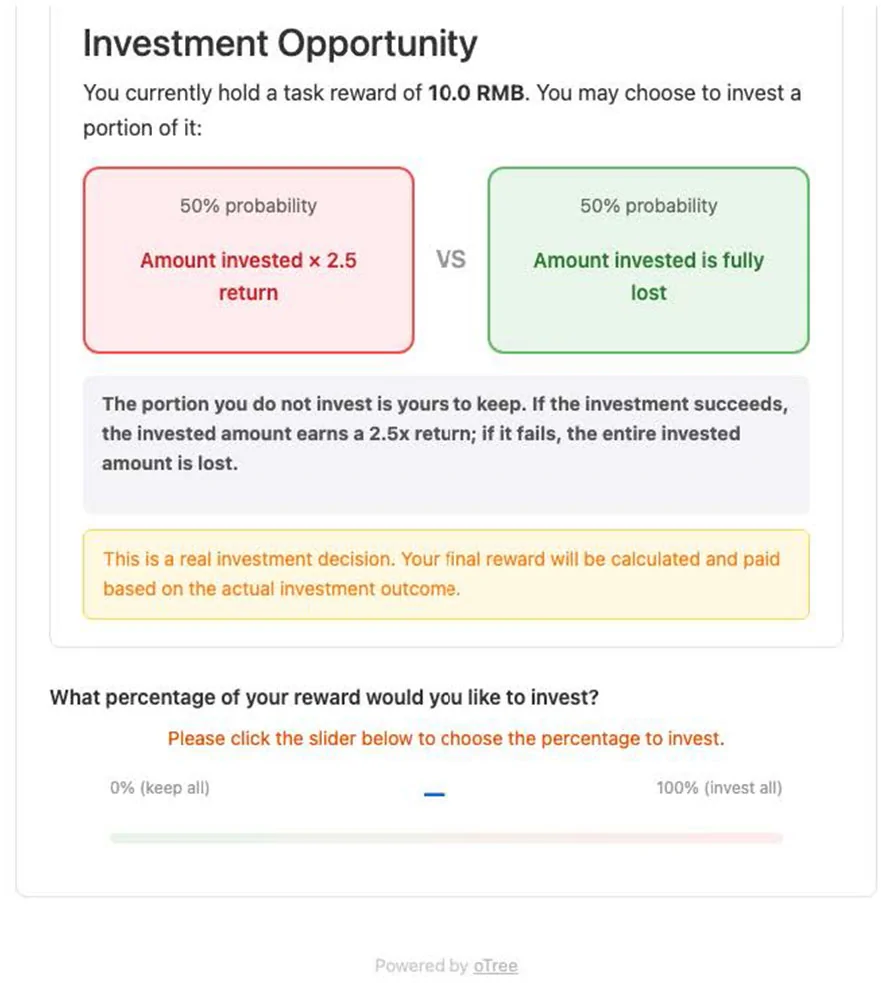
The investment-gamble screen (Gneezy and Potters, 1997) as displayed to participants (English oTree implementation). The endowment of 10 RMB came from the participant's own earnings on the copywriting task; the slider handle was hidden until first interaction to reduce visual anchoring (see Section 3.2 for a caveat on the handle's midpoint resting position); the live-update line below the slider showed the implied invested and retained amounts in real time.

The post-task block administered, in fixed order, the mediator scales (subjective effort, three items adapted from the NASA Task Load Index, Cronbach's α = 0.825; psychological ownership, four items adapted from (Van Dyne and Pierce 2004), Cronbach's α = 0.924); a brief filler task; and the focal investment gamble. Following (Gneezy and Potters 1997), participants chose, on a 0–100% slider, what fraction of the 10 RMB to invest in a one-shot lottery returning 2.5 × with probability 0.5 and 0 otherwise; the unfunded portion was kept. The slider handle was invisible until the first interaction (resting at the scale midpoint), a response could not be submitted before the slider was touched, and an on-screen reminder confirmed that the decision was real-stakes (Figure 2).

The investment gamble was followed, in this order, by a binary hedonic–utilitarian consumption choice, two mental-accounting items (a four-category classification of the earnings on an “earned–windfall” scale and a continuous 1–7 “windfall feel” rating), an attention-check item, the GPS qualitative and staircase risk-preference items (Falk et al., 2018), standard demographic and control items (AI familiarity, stake salience, age, gender, major, monthly disposable income), and finally two manipulation-check items (perceived self-contribution as a 0–100 numeric-entry percentage; perceived AI attribution on a 1–5 Likert scale). The qualitative GPS risk-preference item asked “How willing or unwilling are you to take risks?” on a 0–10 scale; *stake salience* was a single 1–7 item asking “How much do you care about where this 10 RMB ends up?”, anchored from “not at all” to “very much.” The staircase risk-preference task produced a continuous certainty-equivalent index of risk preference that served both as a control variable and as a convergent validity anchor for the investment gamble. Item-level wording for every page of the experiment is given in Appendix 1.

**Figure 3:**
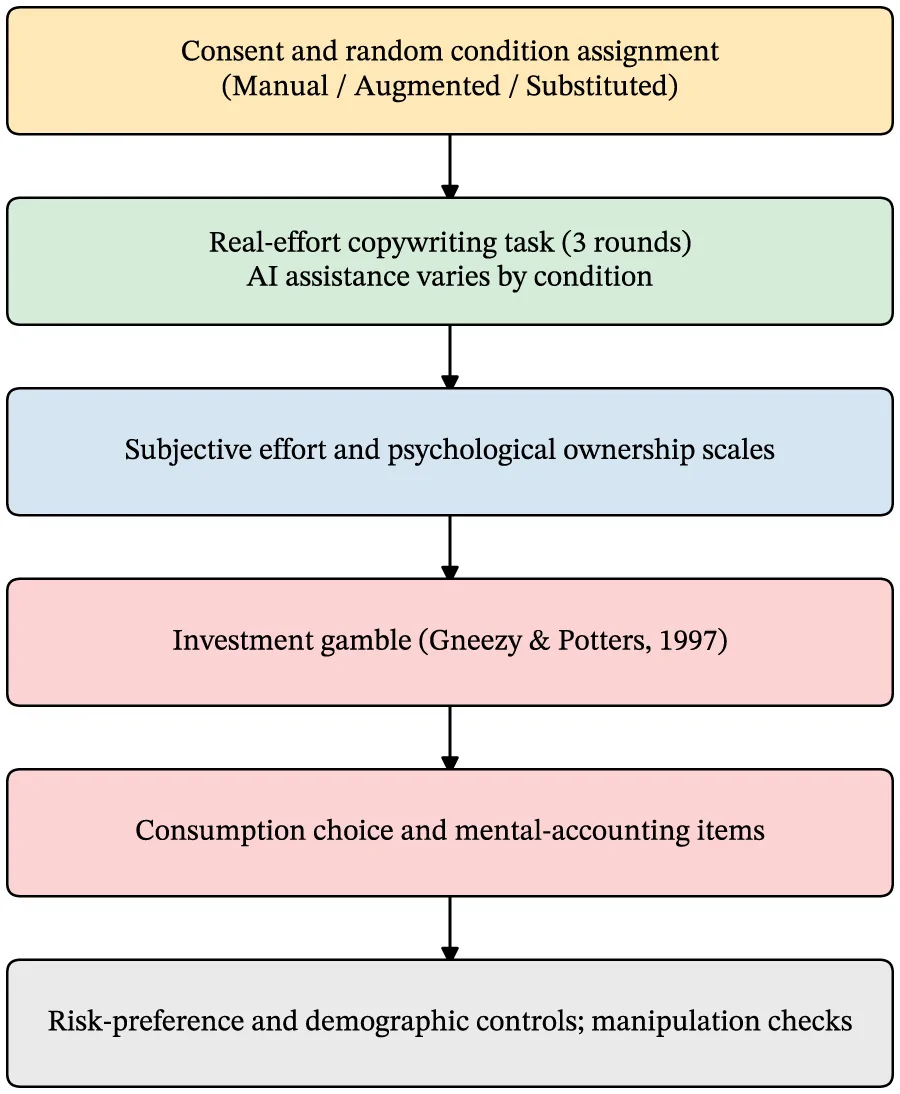
Experimental procedure. After consent and random assignment to one of three AI-assistance conditions, participants completed three rounds of a real-effort copywriting task, then the mediator scales, the incentive-compatible investment gamble, the consumption and mental-accounting items, the risk-preference and demographic controls, and finally the manipulation checks. All within-session pages were administered in fixed order.

Condition assignment was randomized by oTree at the individual level within each session, so sessions are not treatment clusters; each session contained all three conditions in approximately equal proportion (a robustness check clustering standard errors at the session level is reported in Appendix 2). Total session duration averaged approximately 15 min. Figure 3 summarizes the full experimental flow. For analysis, condition was Helmert-coded so that *D*_1_ = (−2/3, +1/3, +1/3) contrasted *Manual* against the average of the two AI-assisted conditions, and *D*_2_ = (0, −1/2, +1/2) contrasted *Augmented* against *Substituted*;^2^ control variables were mean-centered before entry into regression models. Effect sizes are reported as η^2^ for ANOVA (conventional thresholds: 0.01 small, 0.06 medium, 0.14 large) and as Cohen's *d* for pairwise contrasts. Mediation was estimated with a parallel dual-mediator structure and bias-corrected (BC) 95% bootstrap confidence intervals based on 5,000 resamples; minimum detectable effects are reported alongside the primary tests in Section 3.2. Hypotheses, design, exclusion rules, and analysis plan were preregistered at AsPredicted prior to collection of the confirmatory (main) sample; an initial batch of 23 participants had been collected and inspected beforehand to calibrate the power analysis, so the preregistration postdates that pilot batch.^3^ Analysis code is available at the project repository (https://osf.io/kr58v/).

### 2.2 Participants

A total of 202 adults completed the experiment and provided a valid payment account: an initial batch of 23 (the pilot batch inspected before preregistration) and a main confirmatory sample of 179 collected after the analysis plan was frozen. The main sample (*n* = 179: 61 *Manual*, 59 *Augmented*, 59 *Substituted*; all passed the preregistered attention check) is the analytic sample throughout; analyses pooling the pilot batch are reported as robustness checks. Participants were recruited from the participant pool of the Behavioral Science & Digital Agriculture, Rural Areas and Farmers Laboratory at Northeast Agricultural University (Harbin, China). The sample was predominantly female (≈75% female) and majored mostly in economics or management (≈88%), with above-average self-reported AI familiarity (Table 1). Each participant was paid the actual realized outcome of their investment decision by Alipay transfer; the average final payout was approximately 12 RMB (range 0–25 RMB). All participants provided informed consent before beginning the task. The study was approved by the ethics committee of the Experimental Economics Laboratory at Dongbei University of Finance and Economics (Dalian, China; protocol ELAB202603006).^4^

**Table 1:** Sample characteristics by condition (main confirmatory sample, *n* = 179).

Variable	*Manual*	*Augmented*	*Substituted*	*p*
	(*n* = 61)	(*n* = 59)	(*n* = 59)	
	*M (SD)*	*M (SD)*	*M (SD)*	
Age (years)	22.80 (2.80)	23.36 (3.15)	23.73 (2.95)	0.231
Monthly income (1–6)	3.31 (1.09)	3.17 (1.16)	3.42 (1.35)	0.519
AI familiarity (1–7)	4.82 (1.15)	5.20 (1.17)	5.20 (1.06)	0.101
Stake salience (1–7)	4.16 (1.55)	4.20 (1.58)	3.97 (1.75)	0.698
Risk preference (CE, RMB)	96.6 (78.6)	100.1 (86.3)	97.7 (74.6)	0.971
Gender (% female)	82.0%	69.5%	72.9%	0.093

Age, monthly income, and gender are fixed attributes and serve as randomization-balance checks; AI familiarity, stake salience, and risk preference were measured after the manipulation and are descriptive covariates, not balance checks. *p*-values for continuous variables are from one-way ANOVAs on (2, 176) degrees of freedom; the gender *p*-value is from a Pearson χ^2^ test on the 3 (condition) × 3 (gender) contingency table, *df* = 4. All omnibus tests were non-significant. Monthly disposable income is on a 6-band ordinal scale (1= below 1,000 RMB; 6= above 3,000 RMB). AI familiarity, stake salience, and risk preference (CE) were collected after the manipulation and the investment decision; their comparisons describe the analytic sample but cannot certify baseline equivalence, and the higher AI familiarity reported in the AI-assisted conditions may itself be a treatment effect on self-report.

Because the demographic-and-controls block was administered near the end of the session, after the manipulation and all decisions (Appendix 1), only variables that random assignment cannot alter constitute genuine balance checks: age (*F*(2, 176) = 1.48, *p* = 0.23), monthly disposable income (*F* = 0.66, *p* = 0.52), and gender (χ^2^(4) = 7.97, *p* = 0.09) were balanced across conditions. The remaining covariates in Table 1 (AI familiarity, stake salience, and risk preference^5^) were measured after the manipulation and the investment decision, and are therefore post-treatment self-reports rather than baseline attributes. None differed significantly across conditions (AI familiarity *F* = 2.32, *p* = 0.10; stake salience *F* = 0.36, *p* = 0.70; certainty equivalent *F* = 0.03, *p* = 0.97), but the higher reported AI familiarity in the two AI-assisted conditions (4.82 vs. 5.20 and 5.20) could in principle reflect the manipulation rather than chance imbalance. For this reason, the unadjusted models are primary throughout: covariate-adjusted specifications condition on post-treatment variables and are reported for completeness only, and analyses that use these covariates as moderators are labeled exploratory (Appendix 2). Per-condition means and tests are reported in Table 1.

The mediator scales were internally consistent. The three-item subjective-effort composite achieved Cronbach's α = 0.825; the four-item psychological-ownership composite achieved α = 0.924. All four ownership items had corrected item–total correlations between 0.79 and 0.85, and no single-item removal improved α. High α speaks to reliability, not to discriminant validity: the effort and ownership composites correlated at *r*(177) = 0.771 in the analytic sample (full pairwise correlation matrix in Appendix 3), and the two manipulation checks correlated strongly with both composites, so the four perceptual measures may partly index a single perceived-authorship construct. Item content compounds the overlap: one effort item asks how challenging the task felt, which changes objectively across conditions, and one ownership item asks on whom the final headline quality depended, which partly restates the manipulation check. A confirmatory factor analysis on the seven mediator items compared the theorized two-factor model (three effort items; four ownership items, factors free to correlate) against a single perceived-authorship factor. The two-factor model fit significantly better (Δχ^2^(1) = 40.72, *p* < 0.001) and adequately (χ^2^(13) = 30.96, *p* = 0.003; CFI = 0.981, TLI = 0.969, RMSEA = 0.088, SRMR = 0.025), whereas the one-factor model fit poorly (CFI = 0.939, TLI = 0.909, RMSEA = 0.152, SRMR = 0.045); an exploratory maximum-likelihood factor comparison agreed (two-factor AIC 4282.4 vs. one-factor 4318.0). The latent correlation between the two factors, however, was 0.87, so effort and ownership are statistically separable but share roughly three-quarters of their variance; the two mediators should therefore not be read as cleanly distinct mechanisms, and this multicollinearity becomes consequential in the mediation analysis below.

## 3 Results

We report results in the following order: effects on perceptual measures (manipulation checks and mediators), the focal investment decision, mediation through effort and psychological ownership, the mental-accounting classification of the earnings, hedonic consumption, and robustness.

### 3.1 Effects on perceptual measures

**Figure 4:**
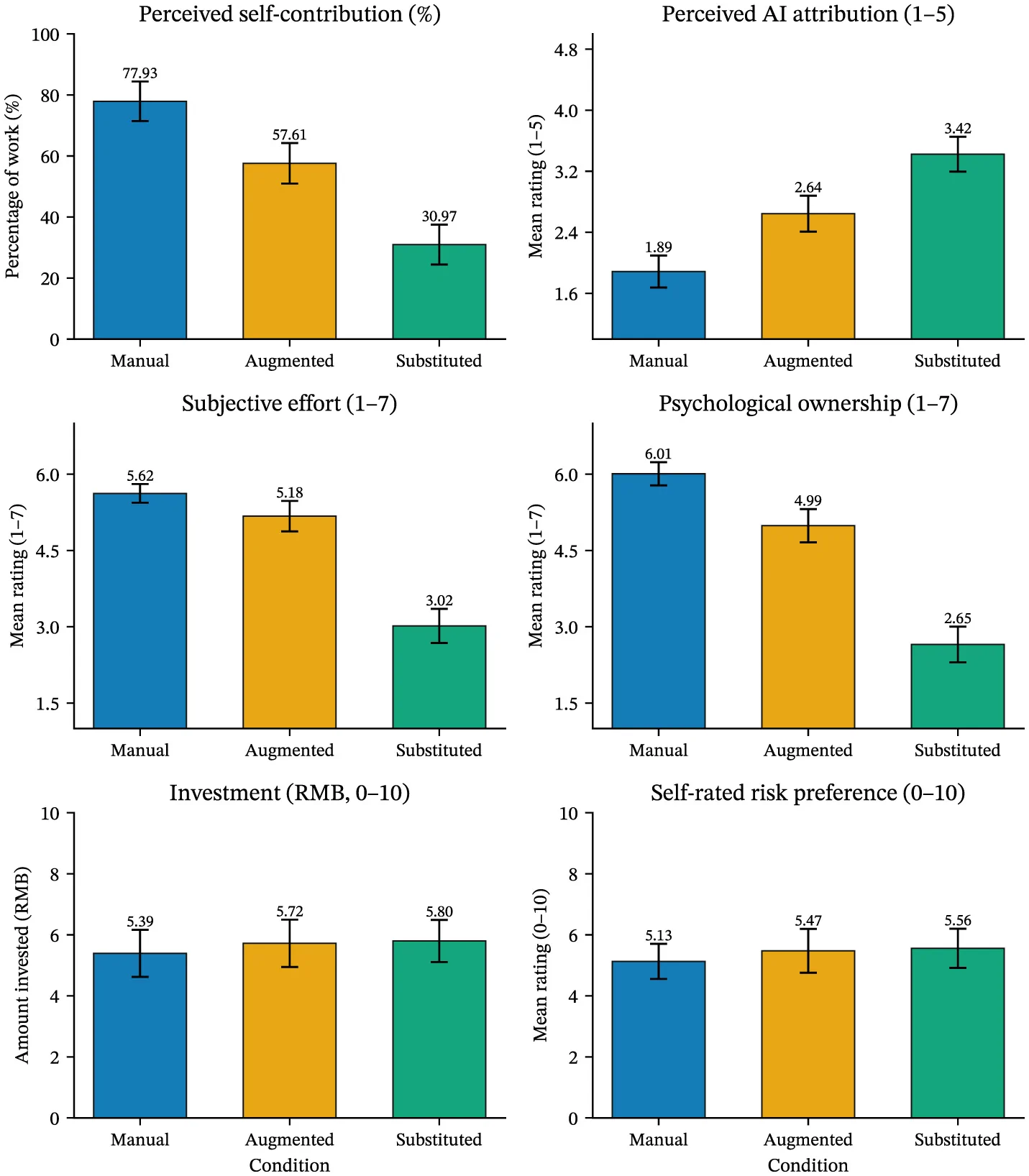
Mean levels of the manipulation checks (top row), mediators (middle row), and risk-decision outcomes (bottom row) by condition. Error bars show 95% confidence intervals. *n* = 179 (main confirmatory sample). Across all six panels, the top four (manipulation checks and mediators) show monotone gradients in the predicted directions (self-contribution, effort, and ownership decreasing with deeper AI assistance; AI attribution increasing) at large effect sizes (η^2^ = 0.35–0.59), whereas the bottom two (investment and self-rated risk) are statistically indistinguishable across conditions.

All four perceptual measures shifted across the three conditions in the predicted direction with large effect sizes (η^2^ = 0.35–0.59; all *F*(2, 176)≥47.28, *p* < 0.001; Figure 4, Table 2). The two manipulation checks tracked the gradient of human contribution: perceived self-contribution declined, and perceived AI attribution rose, with both shifts close in magnitude. All three Tukey post-hoc pairs were significant on each manipulation check (*Manual*–*Augmented* |*d*| = 0.80–0.88; *Augmented*–*Substituted* |*d*| = 0.88–1.05; *Manual*–*Substituted* |*d*| = 1.82–1.86; all *p* < 0.001).

**Table 2:** Manipulation checks and mediator levels by condition (main confirmatory sample, *n* = 179).

Measure	*Manual*	*Augmented*	*Substituted*	ANOVA *F*	Effect size	Trend test
	*M (SD)*	*M (SD)*	*M (SD)*			
Perceived self-contribution	77.93 (25.34)	57.61 (25.47)	30.97 (25.12)	51.89^***^	0.371	−8.47^***^
Perceived AI attribution	1.89 (0.82)	2.64 (0.91)	3.42 (0.88)	47.28^***^	0.349	+7.76^***^
Subjective effort	5.62 (0.72)	5.18 (1.15)	3.02 (1.29)	100.01^***^	0.532	−8.93^***^
Psychological ownership	6.01 (0.89)	4.99 (1.25)	2.65 (1.34)	127.61^***^	0.592	−10.35^***^

^***^p < 0.001. Perceived self-contribution is a 0–100 numeric-entry percentage; perceived AI attribution is on a 1–5 Likert scale; subjective effort and psychological ownership are 1–7 Likert composites. *ANOVA F* is the one-way ANOVA *F* statistic on (2, 176) degrees of freedom; *Effect size* is η^2^, the proportion of total variance in the outcome explained by condition. *Trend test* is the Jonckheere–Terpstra ordered-alternatives *z* statistic; a negative sign indicates a monotone decrease across *Manual*→*Augmented*→*Substituted*.

The two mediator composites tracked the same ordering at larger effect sizes. Subjective effort declined across the three conditions and so did psychological ownership; pairwise Tukey contrasts were significant for every comparison (*d* = 0.94–2.95, all *p* < 0.001) except the adjacent *Manual*–*Augmented* contrast on effort, which was marginal (*d* = 0.47, Tukey *p* = 0.061). Psychological ownership produced the largest single condition effect in the study (η^2^ = 0.592, endpoint *d* = 2.95); ownership rather than effort therefore carries most of the perceptual signal of AI-assisted authorship.

Every measure was *graded* rather than binary: *Augmented* fell strictly between *Manual* and *Substituted* on all four perceptual outcomes, and Jonckheere–Terpstra ordered-alternatives trend tests confirmed strict monotonicity on every measure (all |*z*|≥7.76, *p* < 0.001). The manipulation, therefore, produced a continuous psychological gradient of AI assistance, not a categorical “AI on / off” switch, a precondition for the dual-pathway mediation in Section 3.3.

### 3.2 Effect on the investment decision

The manipulation that moved every perceptual measure (Section 3.1) produced no significant effect on the focal investment decision (Figure 4, bottom row; Table 3, columns 1–2). Mean invested amounts were 5.39 RMB (*SD* = 3.01) in *Manual*, 5.72 (*SD* = 2.97) in *Augmented*, and 5.80 (*SD* = 2.64) in *Substituted* (*F*(2, 176) = 0.34, *p* = 0.72). The means are monotone in the hypothesized direction, with an endpoint contrast of *d* = 0.14 (*Manual* vs. *Substituted*); the same monotone-but-non-significant ordering appears on self-rated risk (5.13, *SD* = 2.25; 5.47, *SD* = 2.76; 5.56, *SD* = 2.47; endpoint *d* = 0.18). The behavioral estimates are therefore directionally consistent with the predictions but imprecisely estimated. The non-significance was robust across non-parametric and ordered-alternatives specifications and reproduced on the derived percent-invested variable and the binary *Manual* vs. AI contrast. Neither *D*_1_ nor *D*_2_ approached significance in the unadjusted (primary) models (Table 3, columns 1 and 3; the covariate-adjusted columns condition on post-treatment variables and are reported for completeness only); H1a and H1b are not supported.

**Table 3:** OLS regressions of investment and self-rated risk on Helmert-coded condition contrasts (main confirmatory sample, *n* = 179).

	Investment (RMB, 0–10)		Self-rated risk (0–10)	
	(1)	(2)	(3)	(4)
*D*_1_ (*Manual* vs. AI-mean)	0.367	0.128	0.386	0.309
	(0.454)	(0.423)	(0.394)	(0.387)
*D*_2_ (*Augmented* vs *Substituted*)	0.076	-0.086	0.085	0.072
	(0.531)	(0.487)	(0.459)	(0.445)
Age		0.163^*^		-0.018
		(0.068)		(0.062)
Monthly income		0.297		0.188
		(0.167)		(0.153)
AI familiarity		0.211		0.194
		(0.176)		(0.161)
Stake salience		-0.219		0.029
	(0.123)		(0.113)
Risk preference (CE)		0.011^***^		0.009^***^
		(0.003)		(0.002)
Constant	5.638^***^	5.636^***^	5.388^***^	5.388^***^
	(0.215)	(0.196)	(0.187)	(0.180)
*N*	179	179	179	179
*R* ^2^	0.004	0.195	0.006	0.104

Standard errors in parentheses. *D*_1_ contrasts *Manual* against the mean of the two AI-assisted conditions; *D*_2_ contrasts *Augmented* against *Substituted*. Columns (1) and (3), without covariates, are the primary specifications; the covariates in columns (2) and (4) were measured after the manipulation (AI familiarity, stake salience, and the certainty equivalent are post-treatment), so the adjusted models are reported for completeness only. Covariates are mean-centerd, so the constant estimates the grand mean in all columns. Risk preference (CE) is the certainty-equivalent index elicited by the GPS staircase task; it is a substantial predictor of both outcomes, consistent with the Gneezy–Potters paradigm capturing genuine individual differences in risk taking, though because the staircase followed the investment decision this association may partly reflect response consistency. Neither *D*_1_ nor *D*_2_ approaches significance on either outcome under either specification. Pooled-sample (*N* = 202) estimates are reported in Appendix 2. ^*^p < 0.05, ^**^p < 0.01, ^***^p < 0.001.

The shape of the allocation distribution qualifies the measure itself. Although the gamble returns 2.5 × at *p* = 0.5, so that the expected value is maximized by investing everything, the mean allocation was 56.4% (*SD* = 28.7), and responses clustered at focal points: 27.4% of participants invested exactly half, 17.9% invested everything, and 5.6% invested nothing. Such marked risk aversion over a stake of roughly one euro, with clumping at 50% and 100%, is consistent with partly heuristic responding, which attenuates any true condition effect; condition-level clustering rates were similar across groups (50%-point: 21–34%; 100%: 16–20%). Two interface details are relevant to the clumping: the slider handle, though invisible until first interaction, rested at the scale midpoint, and no response could be submitted without touching the slider; the 50% mode may therefore partly reflect the initial handle position rather than heuristic responding alone.

Two checks address alternative explanations of the null. (i) Risk preference (the certainty-equivalent measure) significantly predicted the invested amount (*r* = 0.34, *p* < 0.001; full pattern in Appendix 3), indicating that the Gneezy–Potters paradigm (Gneezy and Potters, 1997) captures individual differences in risk taking rather than pure noise. Because the staircase task was administered *after* the investment decision, however, part of this association could reflect a consistency motive in responding, so it supports convergent validity only weakly. (ii) The main confirmatory sample (*n* = 179, α = 0.05) was powered to detect Cohen's *f* = 0.23 at 80%. Two independently computed power bounds, on different scales, both place the observed effect at roughly one-quarter of detectability: on the ANOVA scale, the observed *f* = 0.06 is 26% of the detection threshold; on the regression scale, the observed OLS *D*_1_ coefficient (0.37 RMB) is 29% of the minimum detectable magnitude (≈1.27 RMB) (Lakens, 2013). The corresponding bound computed from the data agrees: the 95% confidence interval for the *D*_1_ coefficient is [−0.53, +1.26] RMB, and a two-one-sided equivalence test rejects condition effects as large as |*d*| = 0.5 (equivalence bounds ±1.44 RMB; larger one-sided *p* = 0.010). The data thus bound the condition effect below a medium size at this stake; they cannot distinguish a true zero from a small effect of the predicted sign. Given the strong condition-to-mediator *a*-paths in Section 3.1 and the null condition-to-outcome relation here, the dual-mediation chain must fail at the mediator-to-outcome stage or through cancellation between pathways, an outcome the parallel-mediation analysis confirms below.

### 3.3 Mediation through effort and psychological ownership

**Figure 5:**
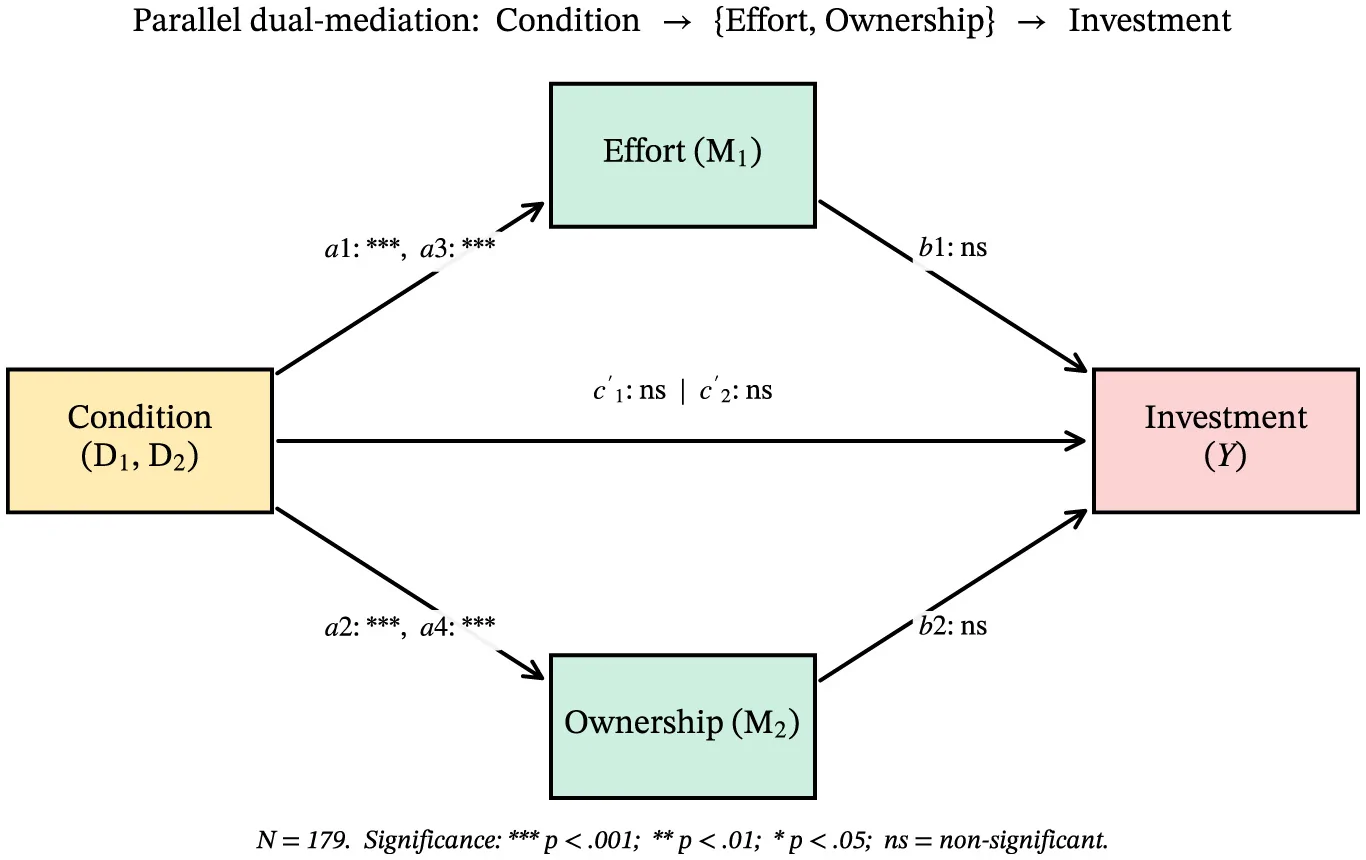
Parallel dual-mediation path diagram. Condition is coded via Helmert contrasts *D*_1_ (*Manual* vs. AI-mean) and *D*_2_ (*Augmented* vs. *Substituted*). Path labels carry only the path name and a significance marker (^***^p < 0.001; *ns* = non-significant); the unstandardized estimates and bootstrap intervals are reported in Table 4. All four *a*-paths (Condition → mediator) are highly significant; both *b*-paths (mediator → investment) are non-significant; all four indirect effects have bias-corrected 95% bootstrap confidence intervals crossing zero. *n* = 179 (main confirmatory sample).

The combination of strong manipulation effects on the mediators and a null effect on the outcome already implies that the mediation will fail; the parallel dual-mediator analysis confirms it with informative structure (Figure 5, Table 4). All four *a*-paths from condition to mediator were large, highly significant, and in the predicted direction: increasing AI assistance reduced both subjective effort and psychological ownership (negative coefficients in Table 4, columns 1–2, reflect the Helmert coding under which *D*_1_ and *D*_2_ both rise with deeper AI involvement). Both *b*-paths from mediator to investment, however, were small and non-significant, and consequently all four bootstrap indirect effects had bias-corrected 95% confidence intervals that crossed zero. Adding the preregistered control block left every interval zero-inclusive. H3 (mediation through subjective effort) and H4 (mediation through psychological ownership) are therefore not supported.

**Table 4:** Parallel dual-mediation of AI-assistance condition on the invested amount through subjective effort (*M*_1_) and psychological ownership (*M*_2_) (main confirmatory sample, *n* = 179; 5,000 bootstrap resamples).

	Effort (*M*_1_)	Ownership (*M*_2_)	Investment (*Y*)
	(1)	(2)	(3)
*D*_1_ (*Manual* vs. AI-mean)	-1.527	-2.188	0.006
	(0.170)	(0.186)	(0.620)
*D*_2_ (*Augmented* vs *Substituted*)	-2.158	-2.335	-0.491
	(0.198)	(0.217)	(0.730)
Effort (*b*_1_)			-0.344
Ownership (*b*_2_)			0.075
			(0.212)
Constant	4.605	4.549	6.880
	(0.080)	(0.088)	(1.050)
*N*	179	179	179
*R* ^2^	0.532	0.592	0.017
Bootstrap indirect effects (5,000 resamples, BC 95% CI)			
*D*_1_: Effort pathway (*a*_1_*b*_1_)	0.525	[−0.227, 1.313]	
*D*_2_: Effort pathway (*a*_3_*b*_1_)	0.742	[−0.318, 1.863]	
*D*_1_: Ownership pathway (*a*_2_*b*_2_)	−0.164	[−1.074, 0.699]	
*D*_2_: Ownership pathway (*a*_4_*b*_2_)	−0.175	[−1.177, 0.736]	

Standard errors in parentheses. Columns (1)–(3) report OLS estimates of the three mediation equations; the indirect-effect panel reports the bootstrap point estimate and bias-corrected (BC) 95% confidence interval based on 5,000 resamples. *D*_1_ contrasts *Manual* against the mean of the two AI-assisted conditions; *D*_2_ contrasts *Augmented* against *Substituted*. The four indirect effects are computed as *a*_*j*_*b*_*k*_ from the equation-by-equation coefficients; none has a 95% BC CI excluding zero. Adding the preregistered control block leaves every interval zero-inclusive. ^*^p < 0.05, ^**^p < 0.01, ^***^p < 0.001.

Two structural features of this null shape the Discussion's interpretation. First, the effort and ownership indirect effects carry *opposite signs*: the effort pathway is non-significantly positive (consistent with the AI-windfall hypothesis), the ownership pathway non-significantly negative (opposite to it). Although neither difference reaches significance, the opposite-sign pattern is the canonical mechanism by which parallel-mediator designs produce aggregate nulls. The two mediators correlate at *r* = 0.771 (Section 2.2); the resulting multicollinearity inflates the standard errors of both *b*-paths and is a plausible co-cause of the cancellation observed here, so the parallel-mediation null cannot be cleanly attributed to opposite-sign mechanisms alone. Second, partialling out the mediators shifts the direct-effect point estimate on *D*_1_ from +0.37 to essentially zero (+0.01, non-significant); after accounting for effort and ownership, no residual direct effect of AI assistance remains, though with all four indirect intervals crossing zero this is a point-estimate decomposition rather than evidence of transmission. An exploratory serial decomposition (effort → ownership → investment) reached the same conclusion: the serial indirect effect was −0.06 for *D*_1_ (BC 95% CI [−0.43, +0.25]) and −0.09 for *D*_2_ (CI [−0.61, +0.36]), with the terminal mediator-to-outcome path remaining null.

### 3.4 Mental-accounting classification of the earnings

**Figure 6:**
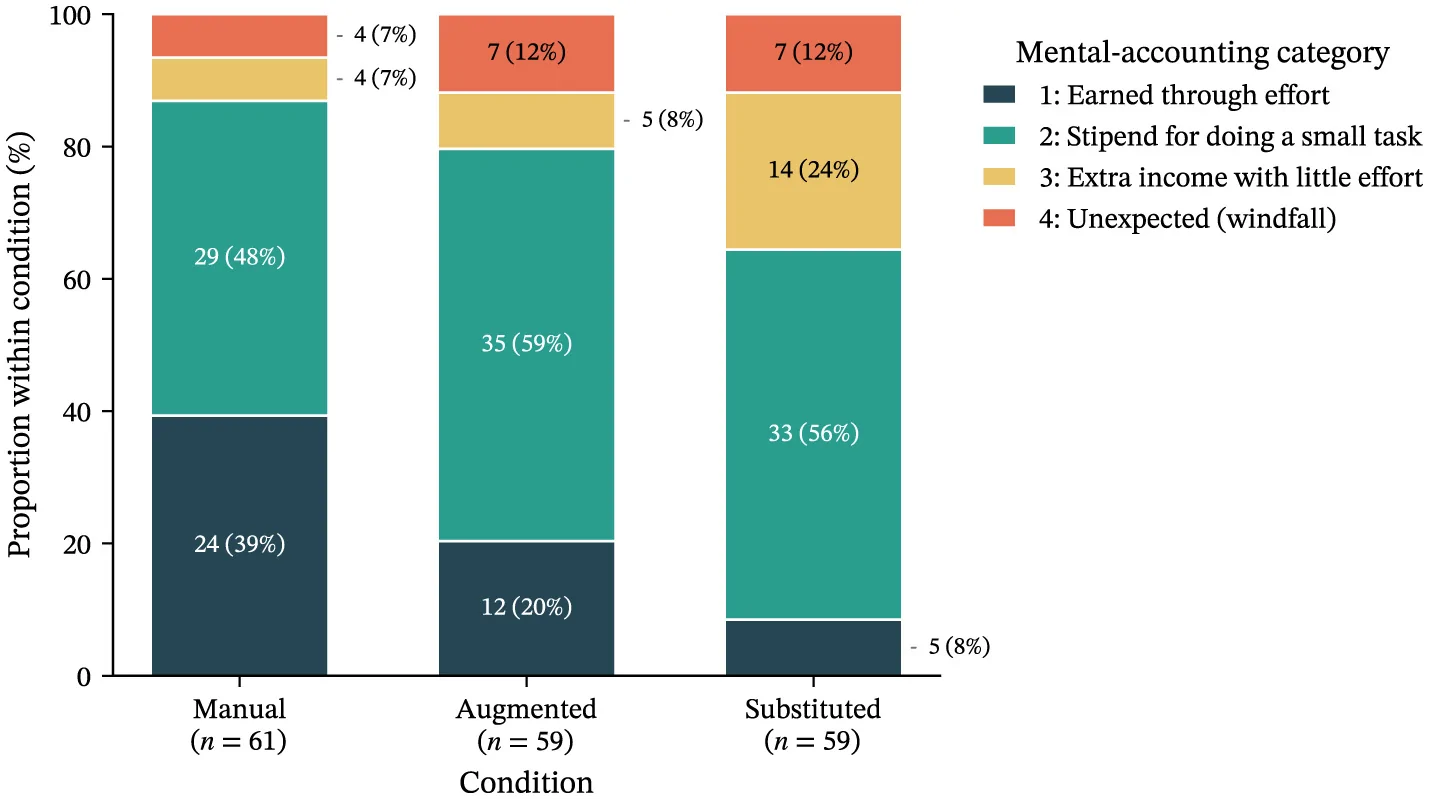
Distribution of responses to the four-category mental-accounting classification item (“Which kind of income does this 10 RMB feel most like?”) across conditions. Categories range from 1 = “earned through effort” to 4 = “unexpected windfall” (full anchors are listed in the legend); every segment is labeled with its raw count and within-condition percentage. An ordered-logit regression yielded a significant shift toward the windfall end of the scale for the AI-assisted conditions: ${\hat{\beta }}_{D_{1}}=1.20$, *OR* = 3.30, *p* < 0.001; Jonckheere–Terpstra trend *z* = 3.81, *p* < 0.001. *n* = 179 (main confirmatory sample).

The categorical classification of the 10 RMB on an earned–windfall scale moved with the manipulation (Figure 6). The proportion of participants choosing the earned-end category fell from 39% in *Manual* to 20% in *Augmented* to 8% in *Substituted*; an ordered-logit regression yielded *OR* = 3.30 for *D*_1_ (*p* < 0.001) and *OR* = 2.04 for *D*_2_ (*p* = 0.046), and the Jonckheere–Terpstra trend test confirmed monotonicity (*z* = 3.81, *p* < 0.001). The parallel descriptive prediction stated in §1 is supported, with an important interpretive caution: three of the item's four category anchors are defined by effort (“earned through my own effort,” “a stipend for doing a small task,” “extra income obtained with little effort”), so the item partly re-measures perceived effort rather than independently capturing mental-account assignment. To that extent the categorical shift is adjacent to the manipulation checks, and its divergence from the flat continuous rating below may reflect item format as much as a cognitive–affective distinction. We therefore do not treat it as an independent behavioral finding. Note also that only 39% of *Manual* participants classified the money as earned through effort, indicating that the flat, non-contingent payment established the earned pole only weakly even in the unassisted condition.

The categorical shift coexisted with a flat continuous rating: the 1–7 “felt-like-a-windfall” item showed no condition difference (*F*(2, 176) = 1.22, *p* = 0.30). One reading is that the manipulation moved *which* mental-accounting bin participants assigned the money to without moving the affective quality of unexpectedness; an alternative, given the anchor wording above, is a format artifact. Both readings are exploratory.

A single item speaks most directly to perceived earnedness: ownership item 2 asked how earned the resulting reward felt (1 = “not earned,” 7 = “fully earned”; Appendix 1, Page 10). Analyzed separately, it showed the largest interpretable gradient in the dataset outside the composites: *M* = 6.05 (*SD* = 1.16) in *Manual*, 4.83 (*SD* = 1.51) in *Augmented*, and 2.83 (*SD* = 1.63) in *Substituted* (*F*(2, 176) = 75.66, *p* < 0.001, η^2^ = 0.46). Yet felt earnedness was only weakly and non-significantly associated with the behavioral outcomes: *r* = −0.08 (*p* = 0.29) with the invested amount and *r* = −0.05 (*p* = 0.48) with the hedonic consumption choice (both negative signs are the direction the windfall account predicts), and *r* = 0.01 (*p* = 0.87) with self-rated risk. The manipulation thus moved perceived earnedness itself strongly, while earnedness in turn barely covaried with spending behavior at this stake.

### 3.5 Hedonic consumption

The binary hedonic–utilitarian consumption choice (hedonic = the beverage voucher; utilitarian = the airtime top-up) did not differ across conditions (χ^2^(2) = 1.16, *p* = 0.56). The preregistered Helmert logit reached the same conclusion: ${\hat{\beta }}_{D_{1}}=0.16$ (*OR* = 1.17, *p* = 0.64), a point estimate whose sign is consistent with H2a but negligible in magnitude, and ${\hat{\beta }}_{D_{2}}=-0.37$ (*OR* = 0.69, *p* = 0.34), whose sign is opposite to H2b. The marginal split was 35% hedonic overall (33% in *Manual*, 41% in *Augmented*, 32% in *Substituted*), so the choice was not at ceiling or floor; the two voucher options were, however, not independently validated as hedonic vs. utilitarian, a limitation of this measure. H2a and H2b are not supported. Re-fitting the parallel mediation model with the hedonic choice as *Y* (linear-probability specification) yielded the following indirect effects: through effort, â*b* = −0.088 for *D*_1_ (BC 95% CI [−0.213, +0.027]) and −0.124 for *D*_2_ (CI [−0.306, +0.040]); through ownership, â*b* = +0.153 for *D*_1_ (CI [−0.001, +0.308]) and +0.163 for *D*_2_ (CI [+0.005, +0.342]). The ownership-pathway estimates are positive (lower ownership predicting a higher probability of the hedonic choice, the direction the windfall account implies), and the *D*_2_ interval nominally excludes zero; because these are exploratory, uncorrected re-analyses of a preregistered-null outcome, we regard the ownership pathway as borderline rather than established, and a powered direct test of it is a natural target for replication. With that caveat, the hedonic choice patterns with the focal investment outcome rather than with the categorical mental-accounting classification.

### 3.6 Robustness

The investment null is robust across alternative analytic choices. Pooling the pre-preregistration pilot batch with the main sample (*N* = 202) leaves every conclusion unchanged: the investment ANOVA remains null (*F*(2, 199) = 0.50, *p* = 0.60; OLS ${\hat{\beta }}_{D_{1}}=0.365$, *p* = 0.39), the perceptual effects remain large (effort *F* = 114.7, ownership *F* = 140.0, both *p* < 0.001), and the categorical mental-accounting shift remains significant (ordered logit *D*_1_: *OR* = 3.16, *p* < 0.001). The null also survives quantile regression across the investment distribution (10th–90th percentiles), with one of the ten coefficients (*D*_1_ at the 25th percentile, +1.30, *p* = 0.022, uncorrected, in the predicted direction) reaching nominal significance; remains null after removing influential observations identified by Cook's *D*; and is unchanged under standard errors clustered at the session level (11 clusters; *D*_1_: *b* = 0.37, clustered *SE* = 0.67, *p* = 0.59), which is conservative given individual-level randomization within sessions. Exploratory moderation analyses found no interaction with AI familiarity, stake salience, risk preference, or gender (all interaction *p*s >0.10; minimum *p* = 0.108); because three of these four moderators were measured post-treatment, these tests are descriptive rather than confirmatory. Full results are reported in Appendix 2.

## 4 Discussion and conclusion

The experiment yields an asymmetric pair of findings. On the perceptual side, the manipulation produced large monotone shifts on every measure: effort, ownership, perceived self-contribution, AI attribution, and, most directly, the feeling that the reward was earned (η^2^ = 0.46 on the single earnedness item). On the behavioral side, no preregistered hypothesis was supported: investment allocation, hedonic-consumption choice, and the four preregistered bootstrap indirect effects through effort and ownership all failed to reach significance, as did self-rated risk preference, a secondary outcome. The behavioral point estimates were directionally consistent with the predictions but small (endpoint *d* = 0.14–0.18, roughly one-quarter of the minimum detectable magnitude), so the data are best read as bounding the perception-to-behavior link below a medium effect at this stake, not as demonstrating its absence. What we label a candidate *perception–behavior dissociation* is therefore a description of this asymmetry, namely strong perceptual movement alongside undetected behavioral movement, rather than an established null.

Four readings are consistent with this pattern; the data cannot yet separate them. *Cue-specificity*: our manipulation targets the earnedness cue of windfall status while leaving unexpectedness and external origin untouched, since in every condition participants knew they would be paid and the money arrived for their task. If windfall *behavior* is driven primarily by affective unexpectedness (Arkes et al., 1994), a manipulation that moves earnedness alone would produce exactly what we observe: strong movement on earnedness reports, none on the flat “felt-like-a-windfall” rating, and no detected behavioral shift. *Cancellation*: the directional predictions were not unambiguous ex ante. If AI-assisted money feels tainted rather than merely unearned, emotional accounting predicts *utilitarian* laundering of the money (Levav and McGraw, 2009), opposing the hedonic prediction; on risk, reduced felt competence could oppose the house-money direction. The opposite-sign indirect effects through effort and ownership in the mediation analysis are suggestive of such cancellation, but testing it requires measuring perceived taint or legitimacy separately from earnedness, which we did not do. *Process-level*: perceptual measures recruit a relatively automatic self-reflective process while economic decisions involve deliberative elaboration (Evans and Stanovich, 2013). *Structural*: the Gneezy–Potters gamble is context-stripped and uncoupled from the labor that produced the endowment, so the result may reflect narrow bracketing (Read et al., 1999). Given the first two readings, the contribution of this study is more safely stated in terms of effort and ownership, which AI assistance reliably and gradedly reduces along with the felt earnedness of the resulting income, than in terms of mental-accounting theory proper, whose windfall construct our manipulation engages only partially.

A methodological implication follows: the within-AI difference between *Augmented* and *Substituted* on subjective effort and ownership, despite both conditions relying on AI-generated text for the final headline, indicates that the psychological signature of AI assistance is not captured merely by whether AI produced the output. Operationalizations of “AI-assisted labor” should attend to the user's perceived depth of participation, not only to whether AI was technically involved.

Several limitations warrant attention. First, the design likely never established a firm “earned” pole from which AI assistance could dislodge the money: payment was flat and non-contingent, the completion fee was disclosed at consent before the manipulation, and only 39% of *Manual* participants classified the money as earned through effort. Relatedly, delegation to AI was assigned rather than chosen, whereas real workers self-select into AI use; both features compress the earned pole. Second, the stake may sit below the threshold at which mental accounting meaningfully engages: 10 RMB is roughly one euro, stake salience sat at the scale midpoint (≈4.1 of 7), the announced quality bonus (up to 50 RMB) was five times the endowment, and the allocation distribution shows the focal-point clumping characteristic of partly heuristic responding. On either route the behavioral null is what one would expect even if the theory is correct, so the experiment constrains the psychology of small assigned-AI stakes, not mental accounting over consequential income. Third, the bonus notice means expected earnings and their subjective variance were not perfectly constant across conditions, and a *Substituted* participant could not appraise the output on which the bonus depended. Fourth, the focal DV is a context-stripped own-account gamble, whereas the earned-endowment effects that replicate most reliably in experimental economics concern allocations to others (Cherry et al., 2002; Jakiela, 2015); a giving or sharing outcome is the better-targeted DV for a follow-up. Fifth, the manipulation centered on a single cognitive-labor type (promotional headline writing), and the one-shot design captures only the immediate psychological response. Finally, the perception–behavior framing is a partly post-hoc reading of a preregistered design whose primary outcome was the investment decision.

Three concrete extensions follow directly. (a) An unearned-endowment control (10 RMB with no task) would establish whether the paradigm responds to endowment source at all, which any dissociation claim requires. (b) Dose–response variation within the AI conditions is informative: in an exploratory analysis within *Augmented*, time on task correlated substantially with psychological ownership (*r* = 0.40, *p* = 0.002) but only weakly and non-significantly with investment (*r* = 0.18, *p* = 0.17), reproducing the study's central asymmetry on continuous within-condition variation. Keystroke-level logs were captured only in *Manual*, so edit-distance measures are unavailable in this dataset; process logging rich enough to support them in all conditions would sharpen this test. (c) Replications should use larger, contingent stakes, chosen rather than assigned delegation, consumption stimuli independently pre-tested as hedonic vs. utilitarian, and outcome domains with reliably documented earnedness effects (giving, sharing, earmarking), alongside AI–labor pairings beyond copywriting and longitudinal designs that capture habituation.

AI assistance reliably changed how labor and its income feel in this sample: participants doing AI-assisted work reported gradedly less effort, less ownership, and less earned income, with the largest effects in the study. At this small, flat-paid stake, we did not detect a corresponding change in how the income was spent, and the behavioral estimates, which are directionally consistent with the predictions but roughly one-quarter of the minimum detectable magnitude, bound rather than refute the downstream consequences. The graded manipulation protocol and the quantitative bounds on the null provide a reproducible starting point for follow-up work asking when, at what stakes, and for whom AI-related changes in felt effort and ownership translate into economic decisions.

## Data Availability

The raw data supporting the conclusions of this article will be made available by the authors, without undue reservation.

## Footnotes

1 Headline generation in the *Augmented* and *Substituted* conditions used DeepSeek-V3, accessed in real time at request through the official DeepSeek API.

2 Helmert coding represents the three-level condition variable as two orthogonal numeric contrasts. The chosen weights make ${\hat{\beta }}_{D_{1}}$ test the *Manual* vs. AI-pooled contrast (corresponding to H1a) and ${\hat{\beta }}_{D_{2}}$ test the *Augmented* vs. *Substituted* contrast (corresponding to H1b); the orthogonality (∑*D*_1_*D*_2_ = 0) ensures the two coefficients can be interpreted independently, without the inflation of standard errors that follows from non-orthogonal coding. Compared with dummy coding, the Helmert weights map a priori hypotheses onto regression coefficients directly, avoiding post-hoc linear combinations.

3 AsPredicted #282,931, “Effect of AI involvement on risk decisions with earned income, online experiment” (https://aspredicted.org/uc2pr4.pdf). The single preregistered exclusion criterion was failure of an attention-check item; all completers passed this check, so no participants were excluded under this rule. The main confirmatory sample (*n* = 179), collected entirely after the analysis plan was frozen, is the analytic sample throughout; analyses pooling the pilot batch (*N* = 202) are reported as robustness checks (Section 3.6).

4 Northeast Agricultural University does not maintain a standing institutional review board for behavioral-science research; ethical review and approval were therefore obtained, by prior arrangement, from the partner laboratory.

5 Risk preference is operationalized as the certainty equivalent of a 50/50 lottery for 300 RMB, elicited with the GPS staircase procedure Falk et al., 2018. The staircase begins with the active interval [0, 320] RMB; at each of five rounds, the participant chooses between the lottery (50% chance of 300 RMB, 50% chance of nothing) and a sure payment equal to the midpoint of the active interval. A “lottery” choice raises the lower bound of the active interval to that midpoint; a “sure payment” choice lowers the upper bound to that midpoint. After the fifth round, the certainty equivalent is recorded as the midpoint of the final interval, with resolution ±5 RMB.
