# Supplementary material for: Does AI-assisted labor change how earnings are spent?
Source: Front Psychol. 2026 Sep 3;17:1877013. doi: 10.3389/fpsyg.2026.1877013 (PMC13582443; doi:10.3389/fpsyg.2026.1877013)
Supplement: Supplementary file 1 [file Data_Sheet_1.pdf]

Supplementary material

Does AI-assisted labor change how earnings are spent?

Jinru Zong[1] and Zhuo Lyu[2,*]

[1]International Cultural and Educational College, Northeast Agricultural University, Harbin, China

[2]School of Economics and Management, Northeast Agricultural University, Harbin, China

[*]Correspondence: Zhuo Lyu, lvzhuo@neau.edu.cn

Section and figure/table numbers of the form “Section 2.1,” “Figure 2,” or “Table 4” refer to the main article; supplementary figures herein are numbered S1, S2.

1 Page-by-page content of the experimental flow

This appendix documents the full participant-facing content of every page in the order it was administered. All wording is translated from the Chinese-language oTree templates; the original templates are available in the project repository (`https://osf.io/kr58v/`).

A.1 Setup

Page 1: Introduction (consent). A welcome message tells participants that the study is an academic investigation of “creative work and decision behaviour,” takes about 15–30 min, and pays a base fee of 10 RMB on completion (real money). Participant rights are listed:

- participation is voluntary;
- participants may withdraw at any time;
- all responses are kept confidential and anonymised.

The page closes with an explicit consent statement: “Clicking ’Next’ indicates that you have read and agreed to the above and voluntarily consent to participate.”

Page 2: Task instruction. Tells participants that they will write three short headlines (one per product, $\leq$ 20 Chinese characters each) and lays out the condition-specific procedure:

- *Manual*: read the product, write a headline, submit.
- *Augmented*: read the product, type three keywords; the AI returns three candidates; select one, optionally edit, submit.
- *Substituted*: read the product, click “auto-generate,” confirm and submit.

A standing notice tells participants that the top 1% of headlines will receive an additional 50 RMB and the next 9% will receive 20 RMB; this notice was constant across conditions.

A.2 Real-effort task block (three rounds)

Pages 3, 5, 7: Copywriting task (rounds 1–3). Each round shows one of three products (Bluetooth earphones, vacuum flask, portable charger) with brand, descriptive specifications, and retail price. A condition-specific badge above the input area reinforces the source attribution: "This round is completed by you alone" / "... by you and the AI assistant together" / "... by the AI assistant." Figure S1 shows the three condition-specific layouts of this page.

Pages 4, 6, 8: Round-feedback screens. A short confirmation page (auto-advancing after 4 s) showing "Round k completed!", the headline that the participant submitted, and the condition-specific attribution message:

- *Manual*: "This round was completed by you alone."
- *Augmented*: "This round was completed by you and the AI assistant together."
- *Substituted*: "This round was completed by the AI assistant for you."

A.3 Reward notification and post-task block

Page 9: Reward notification. "All tasks completed. You earned 10 RMB." The screen shows the 10 RMB amount in large type and the condition-specific framing:

- *Manual*: "You completed the entire task through your own effort and earned 10 RMB."
- *Augmented*: "You completed the entire task with the AI assistant's help and earned 10 RMB."
- *Substituted*: "The AI assistant completed the entire task for you, and you received 10 RMB."

The page ends by telling participants that they will next decide how to handle these earnings.

Page 10: Subjective effort and psychological ownership scales (TaskFeelings). Seven 1–7 Likert items, all referring to the just-completed copywriting task. The three subjective-effort items asked:

- how much effort the participant put in (1 = "very little"; 7 = "a great deal");
- level of concentration and engagement (1 = "not at all engaged"; 7 = "highly engaged");
- how challenging the task felt (1 = "not challenging"; 7 = "very challenging").

The four psychological-ownership items asked:

- who the final headline quality depended on (1 = "entirely the AI"; 7 = "entirely me");
- how earned the resulting reward felt (1 = "not earned"; 7 = "fully earned");
- how much the headline felt like the participant's own work (1 = "not my work"; 7 = "entirely my work");
- the extent of being an active driver versus a passive observer (1 = "observer"; 7 = "driver").

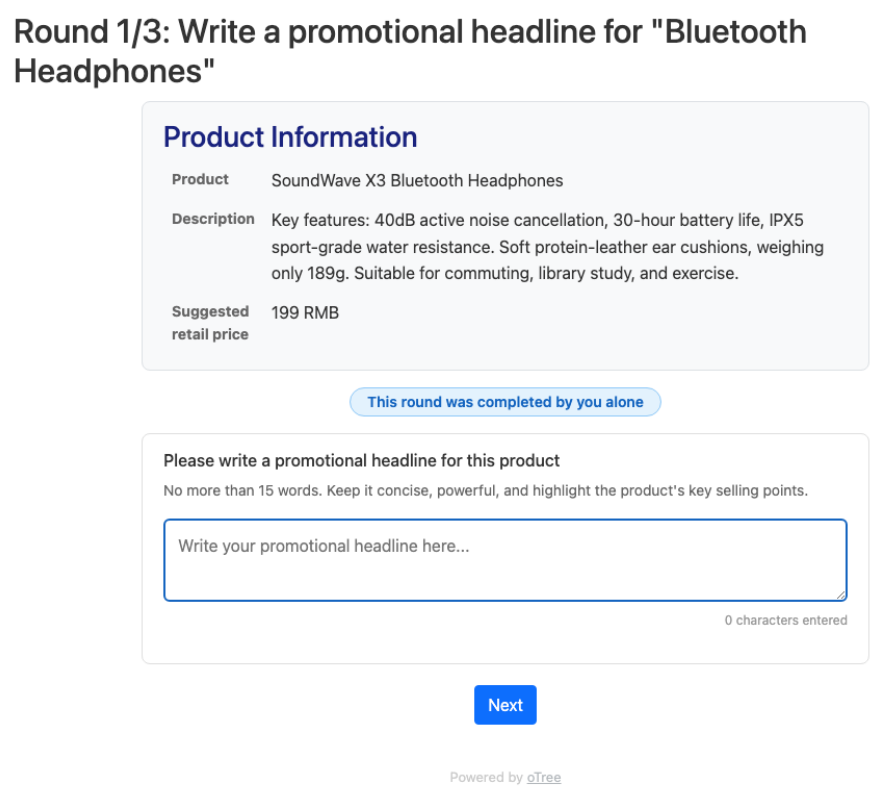

(a) *Manual: free-form text input.*

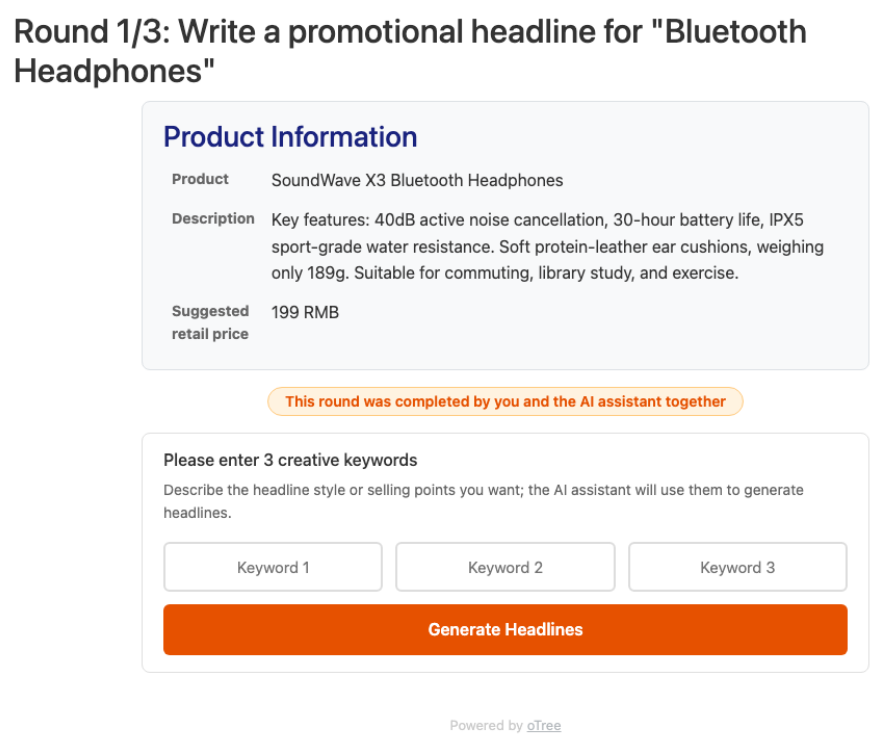

(b) *Augmented: keywords → AI candidates → select & edit.*

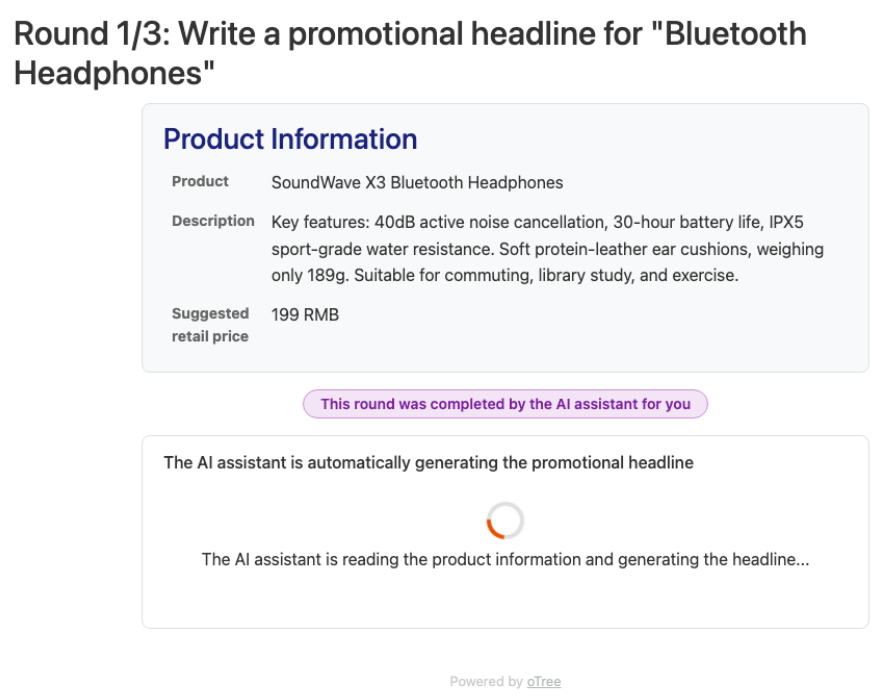

(c) *Substituted: a single "auto-generate" button; participant only confirms.*

Figure S1: Screenshots of the copywriting-task screen across the three conditions (English oTree implementation). The product card and the condition-specific attribution badge above the input area are identical in structure across conditions; only the input mechanism differs.

Page 11: Filler task. A single forced-choice item asking "Which of these three slogans appeals to you most?" across three unrelated slogans, with a 30-s timeout. The page is included to introduce a brief perceptual buffer between the subjective measures and the subsequent decision tasks.

Page 12: Investment gamble. The participant is endowed with the 10 RMB just earned and chooses, on a 0–100% slider, what fraction to invest in a 50/50 lottery returning 2.5× or 0. The slider handle is invisible until the first interaction to prevent visual anchoring, but rests at the scale midpoint; the response field is only populated once the slider is touched, and a live-update line below the slider shows the implied invested and retained amounts. An explicit on-screen reminder confirms that the decision is real-stakes and that the final payout is determined by the actual outcome (Figure 2 of the main article; see Section 2.1).

Page 13: Hedonic vs. utilitarian consumption choice and mental-accounting items. Four items follow the investment decision, in fixed order:

(a) Hypothetically, if the 10 RMB earnings were converted to one of two equal-value vouchers, which would the participant prefer: a Luckin Coffee/Mixue beverage coupon (hedonic) or a mobile-phone airtime top-up (utilitarian)?

(b) Four-category mental-accounting classification: which of the following best describes this 10 RMB?

1. income earned through my own effort
2. a stipend for doing a small task
3. extra income obtained with little effort
4. an unexpected small windfall / red-envelope

(c) A continuous 1–7 rating of the extent to which the money "feels like an unexpected gain."

(d) An attention-check item that asked the participant to select "agree."

Page 14: GPS qualitative risk-preference item. A single Falk et al. (2018) GPS item: "Overall, how willing or unwilling are you to take risks?" on a 0–10 scale (0 = "not at all willing"; 10 = "very willing").

Page 15: GPS staircase certainty-equivalent task. Five sequential binary lottery choices that converge on the participant's certainty equivalent of a 50/50 lottery for 300 RMB. At each step the participant chooses between "Lottery: 50% chance of 300 RMB, 50% chance of nothing" and "Sure payment of X RMB," where the offered X is updated by binary search on the interval $[0, 320]$ RMB. After five rounds the certainty equivalent is recorded.

Page 16: Demographics and controls.

- AI familiarity: 1–7 Likert (“how often do you use ChatGPT / DeepSeek / Wenxin?”).
- Stake salience: 1–7 Likert (“how much do you care about where this 10 RMB ends up?”).
- Age: numeric, 16–80.
- Gender: male / female / other.
- Major: Economics/Management, Humanities/Law, STEM, Medicine, Other.
- Monthly disposable income: six ordinal bands from below 1,000 RMB to above 3,000 RMB.

Page 17: Manipulation checks. Two items targeting the manipulation. MC1: “In the task you just completed, what percentage of the work would you say was done by yourself?” as a 0–100 numeric-entry percentage (no pre-filled default). MC2: “Whose effort was the reward primarily produced by?” on a 1–5 ordinal scale anchored from “entirely myself” (1) to “entirely the AI assistant” (5).

Page 18: Payment and Alipay account. A summary screen lists the base reward, the chosen invested fraction and amount, the realised lottery outcome (win / lose / no investment), and the final payout. The participant is then asked to enter and reconfirm an Alipay account; payment is committed within seven business days.

Page 19: Debriefing. A final thank-you screen reaffirming the seven-day payout schedule and confirming that the bonus rewards (top 1% / next 9% on slogan quality) will be paid after the full data-collection period closes.

2 Robustness checks

This appendix reports the robustness analyses summarised in Section 3.6 of the main article.

Subset analyses. The initial 23-participant batch was inspected before the sample-size decision for the main collection and precedes the preregistration; the main-collection sample ($n = 179$) is therefore the primary confirmatory sample throughout the paper, and the pooled sample ($N = 202$) serves as a sensitivity analysis. In the pooled sample, the H1 investment ANOVA remained null ($F(2, 199) = 0.50$, $p = 0.60$; OLS $\hat{\beta}_{D_1} = 0.365$, $p = 0.39$), the perceptual-measure effects remained large (effort: $F = 114.7$; ownership: $F = 140.0$; both $p < 0.001$), and the categorical mental-accounting shift remained significant (ordered logit D_1: $OR = 3.16$, $p < 0.001$). The pilot subset alone showed the same qualitative pattern.

Session-clustered standard errors. Randomization was at the individual level within sessions, so sessions are not treatment clusters; as a conservative check, refitting the unadjusted H1 OLS with standard errors clustered by session (11 clusters) left the conclusions unchanged: $\hat{\beta}_{D_1} = 0.37$ (clustered $SE = 0.67$, $p = 0.59$), $\hat{\beta}_{D_2} = 0.08$ (clustered $SE = 0.67$, $p = 0.91$), versus conventional SEs of 0.45 and 0.53.

Manipulation checks within the Manual condition. Although no AI was present in *Manual*, mean perceived self-contribution was 77.9% and mean AI attribution 1.89 of 5. The within-*Manual* distributions clarify this: on the self-contribution item (a 0–100 numeric-entry percentage with no pre-filled default), the median was 80%, 36% of participants answered exactly 100%, and 6 of 61 answered 50%; on the AI-attribution item, 50 of 61 participants chose the two lowest categories ("entirely myself" or next), 8 chose the midpoint, and 3 chose category 4. The sub-ceiling means thus reflect a mixture of scale-use conservatism and a minority of midpoint responses rather than a uniform belief that AI contributed.

Quantile regressions on the investment distribution. We refit the H1 OLS at the 10th, 25th, 50th, 75th, and 90th percentiles of the investment distribution. One of the ten conditional-quantile coefficients reached nominal significance: D_1 at the 25th percentile ($\hat{\beta}_{D_1} = +1.30$, $p = 0.022$, uncorrected), in the direction predicted by the AI-windfall hypothesis; no other coefficient approached significance (all remaining $p \geq 0.36$), and the median and upper-quantile estimates were essentially zero. Given ten uncorrected tests, we read this as consistent with the overall null rather than as evidence of a lower-tail effect.

Moderation by individual-difference measures (exploratory). Because AI familiarity, stake salience, and risk preference were measured after the manipulation, only the gender moderation is strictly confirmatory; all four tests are reported here as exploratory. None of the four individual-difference measures interacted with the condition effect on investment. With investment regressed on D_1, D_2, the moderator, and its interactions with both contrasts, no individual interaction coefficient reached significance for AI familiarity, stake salience, risk preference, or gender (minimum interaction $p = 0.108$). Median-split subgroup ANOVAs by AI familiarity, stake salience, and risk preference all returned $F < 1$ within each half.

Influence diagnostics. On the H1 OLS model with full controls, Cook's D identified 8 cases above the $4/n$ threshold, none above 1. Refitting after removing the top-five most-influential cases left the condition coefficients still null ($\hat{\beta}_{D_1} = 0.01$, $p = 0.98$; $\hat{\beta}_{D_2} = -0.33$, $p = 0.48$); the qualitative pattern is unaffected by influential observations.

3 Pairwise correlations among key variables

Figure S2 shows the lower-triangle pairwise Pearson correlation matrix among all key analytic variables. Two patterns relevant to the analyses in the main text are visible at a glance: the strong inter-correlation between the two mediators (effort $\times$ ownership), which drives the multicollinearity discussed in Section 3.3 of the main article, and the robust correlation between risk preference (the certainty-equivalent measure) and the investment allocation, which supports the convergent validity of the Gneezy–Potters paradigm in this sample. Both mediators correlate with investment at low magnitudes, consistent with the null indirect effects reported in Table 4 of the main article.

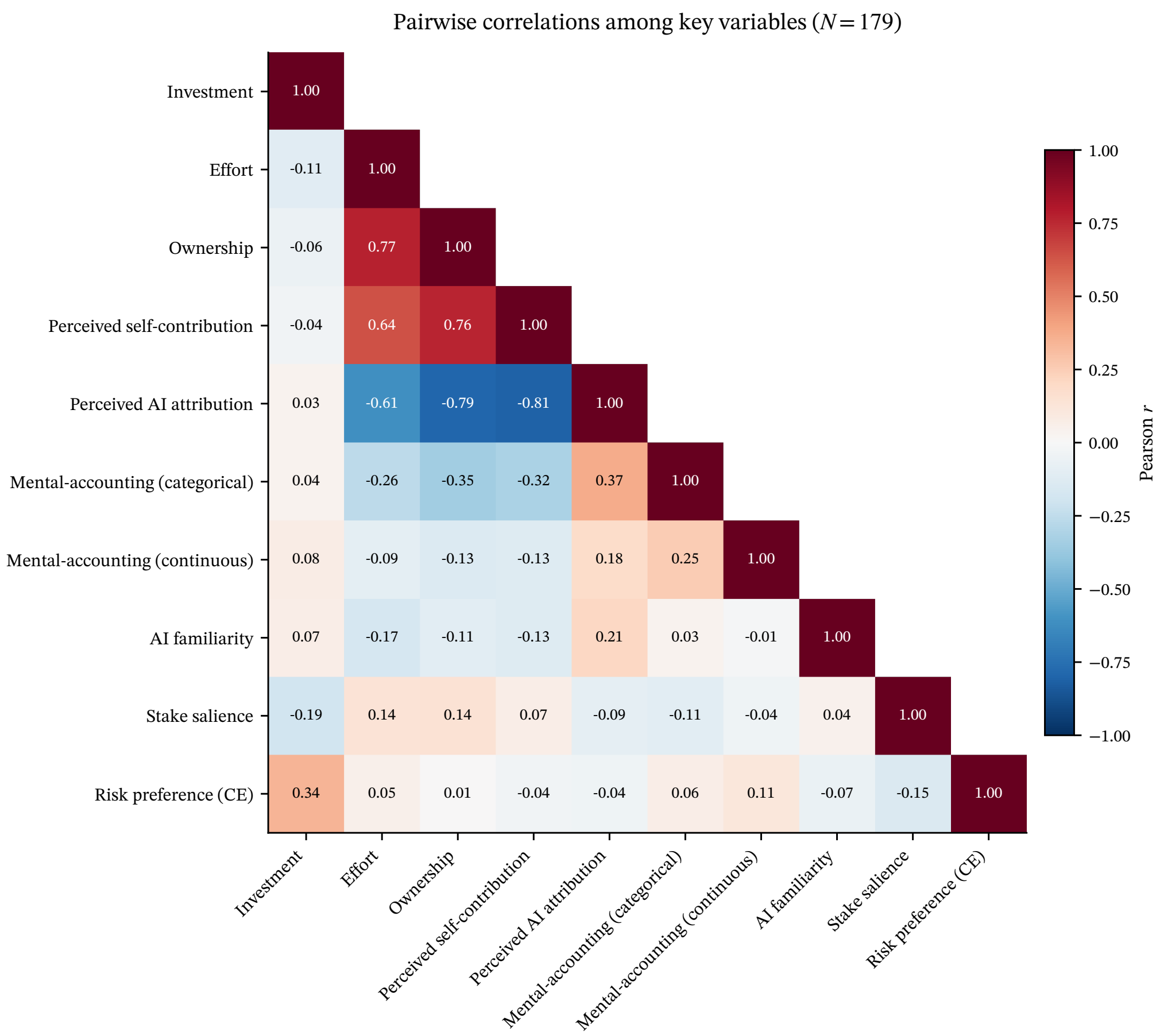

Figure S2: Lower-triangle pairwise Pearson correlations among key variables ($n = 179$, main confirmatory sample).
